# High Proportion of HIV Serodiscordance among HIV-Affected Married Couples in Northern Vietnam

**DOI:** 10.1371/journal.pone.0125299

**Published:** 2015-04-21

**Authors:** Ikumi Sawada, Junko Tanuma, Cuong Duy Do, Tra Thu Doan, Quynh Phuong Luu, Lan Anh Thi Nguyen, Tuong Van Thi Vu, Tuan Quang Nguyen, Naho Tsuchiya, Teiichiro Shiino, Lay-Myint Yoshida, Thanh Thuy Thi Pham, Koya Ariyoshi, Shinichi Oka

**Affiliations:** 1 Department of Clinical Tropical Medicine, Institute of Tropical Medicine, Graduate School of Biomedical Science, Nagasaki University, Nagasaki, Japan; 2 AIDS Clinical Center, National Center of Global Health and Medicine, Tokyo, Japan; 3 Department of Infectious Diseases, Bach Mai Hospital, Hanoi, Vietnam; 4 Laboratory for Molecular Diagnostics, Department of Immunology and Molecular Biology, National Institute of Hygiene and Epidemiology, Hanoi, Vietnam; 5 Microbiology Department, Bach Mai Hospital, Hanoi, Vietnam; 6 Infectious Diseases Surveillance Center, National Institute of Infectious Diseases, Tokyo, Japan; 7 Department of Pediatric Infectious Diseases, Institute of Tropical Medicine, Nagasaki University, Nagasaki, Japan; University of Athens, Medical School, GREECE

## Abstract

**Introduction:**

Little is known about the state of HIV transmission among married couples in Vietnam. This study aims to clarify HIV serostatus in this group and elucidate risk factors for intra-marital HIV transmission.

**Methods:**

In 2012, we enrolled a group of HIV-positive married men registered at the HIV outpatient clinic of a referral hospital in northern Vietnam, along with their wives. Sociodemographic, behavioural and clinical data were collected from men and wives. HIV serodiscordant couples were followed until March 2014 to determine seroconversion rate. A phylogenetic analysis was performed based on *env* V3 sequence to detail cluster formation among men.

**Results:**

Of the 163 HIV-positive men enrolled in the study, 101 (62.0%) had wives testing HIV-negative. Half of men reported injecting drug use (IDU) as a likely transmission route. Couples reported a high incidence of unprotected sexual intercourse prior to diagnosis; the median (inter quartile range) was 4 (4–8) times per month. Only 17 couples (10.4%) reported using condoms during at least half these instances. Multivariable analysis revealed IDU history among men was independently associated with HIV-negative wives (adjusted OR 0.31; 95% CI 0.10–0.95, p=0.041). Phylogenetic analysis of 80 samples indicated CRF01_AE. Of these, 69 (86.3%) clustered with IDU-associated viruses from Vietnam. No HIV seroconversion was identified during a follow-up of 61 serodiscordant couples, with 126.5 person-years of observation during which HIV-infected men were on antiretroviral drug therapy (ART).

**Conclusion:**

High HIV serodiscordance was observed among HIV-affected married couples in northern Vietnam. A large number of at-risk wives therefore remain HIV-negative and can be protected with measures including proper use of ART if couples are made aware of the serodiscordance through screening.

## Introduction

In Vietnam, the HIV epidemic first surfaced among injecting drug users (IDUs) and female sex workers (FSWs) in the 1990s, after the virus was introduced to southern Vietnam through heterosexual contact [1,2]. In 2012, the epidemic reached an estimated prevalence among the adult population of 0.47%, up from of 0.20% in 2000 [3]. Since IDUs in Vietnam are almost exclusively male, the epidemic initially exhibited male-indexed characteristics; more than 80% of reported HIV cases in the 1990s were among men [1,4]. HIV spread rapidly in the IDU population, particularly in northern Vietnam, where estimated HIV prevalence among IDUs in 2009 was in the range of 21% to 56% [5]. A series of HIV CRF01_AE epidemics consisting of three clusters have been reported: a heterosexual-transmission associated cluster in southern Vietnam, an IDU associated cluster in southern Vietnam, and an IDU associated cluster in northern Vietnam, spreading in that order [2].

Of note, the spread of HIV to the general population was much slower than in Thailand and Cambodia in the 1990s [6]. In Thailand, a large bridging population of male sex-worker clients contributed to a rapid spread to the general population. As evidence, HIV prevalence among pregnant women in Thailand quadrupled within the first five years of the epidemic [6,7].

Conversely, the spread of HIV to women in the general population of Vietnam was surprisingly slow. In 2012, a decade after the start of the epidemic, the male-to-female infection ratio remained as high as 2.5, only slightly lower than the ratio of 3.2 in 2000 [3,6]. Most HIV-positive women in Vietnam are neither FSWs nor IDUs; instead they contracted HIV from a male sex partner, himself either an IDU or FSW client [8,9]. This intimate partner transmission has been attributed to a lack of awareness of infection risks, low condom adherence among fixed partners, and male dominance in sexual decision making [9–11]. As such, the threat of HIV spread into the general population in Vietnam remains.

To further control the HIV epidemic in Vietnam, intimate partner transmission from HIV-positive men to wives must be better understood. However, there have been no studies comprehensively investigating HIV-affected couples in Vietnam. Most research on HIV prevention has focused on high-risk groups such as IDUs, FSWs, or men who have sex with men (MSM). The objective of this study is to reveal the sociodemographic and clinical characteristics of HIV-affected married couples in which the husband is HIV-positive, to elucidate factors determining HIV status among wives and to determine incidence of HIV transmission after HIV diagnosis.

## Materials and Methods

### Study population

A hospital-based cohort study was conducted at the Department of Infectious Diseases of Bach Mai Hospital (BMH) in Hanoi, the largest referral hospital in northern Vietnam. The study team evaluated patient records to facilitate approaches to HIV-positive married men registered at the clinic between 26 September 2009 and 31 December 2011, along with these patients’ wives. This includes legally married men and men considered married in the Vietnamese social context. The following cases were excluded from the analysis: patients who did not agree to bring their wives for HIV screening; patients who rejected participation; patients who had been married after their HIV diagnosis; and patients whose clinical information at the time of diagnosis was unavailable.

### Data collection

From 1 January to 31 December 2012, all eligible couples were independently invited to answer a standardized questionnaire written in Vietnamese. The questionnaire included questions on age, occupation, educational attainment, marital status, number of children, desire to conceive, sexual behaviours before and after HIV diagnosis, possible risks of HIV transmission, history of contact with FSWs, and history of genital ulcers. Those reporting an IDU history received further questions regarding use of injection equipment (sharing) and the age at which they both initiated and (if applicable) terminated drug use. A trained nurse was assigned to assist participants having trouble with the questionnaire. Clinical information collected from medical records at the initial visit included date of HIV diagnosis, baseline CD4+ T cell count (cells/μl), baseline viral load (log_10_ copies/ml), anti-hepatitis C virus antibody (HCV Ab), hepatitis B surface antigen (HBsAg), date of antiretroviral treatment (ART) initiation, and clinical stage at the time of HIV diagnosis. Evaluation of clinical stage was based on Vietnam’s national guidelines for HIV/AIDS diagnosis and treatment [12]. Later, between 1 January and 31 March 2014, all eligible HIV serodiscordant couples in the study were invited to be tested for HIV serology and asked to complete a follow-up questionnaire regarding their sexual behaviour in 2013.

### HIV screening

Wives previously testing HIV-negative as well as wives not yet tested for HIV serology were tested to determine initial HIV serostatus at the time of enrolment. The tests were performed at the Microbiology Department at BMH. An HIV-positive diagnosis was made when serum tested positive in the screening test (Alere Determine HIV-1/2, Alere Medical Japan, Japan) and two different anti-HIV antibody enzyme-linked immunosorbent assays following the official diagnostic protocol of the Ministry of Health of Vietnam [12]. Either of the two following kits were used: Genscree ULTRA HIV Ag-Ab, Bio-Rad Laboratories, France; Murex HIV Ag/Ab Combination, DiaSorin Dartford, UK; Serodia HIV-1/2, Fujirebio Inc., Japan; and Roche Elecsys HIV Combi, Roch Diagnostics, Germany.

### Sequence and phylogenetic analyses

Approximately 6 millilitres of blood was collected from HIV-positive men using BD Vacutainer CPT Tube (BD, USA) at the HIV clinic of BMH. Buffy coats were separated using centrifugation, stored at -80°C at the Microbiology Department and then transported to the Department of Immunology and Molecular Biology at the National Institute of Hygiene and Epidemiology in Hanoi for follow-up analysis.

To conduct the phylogenetic analysis of HIV-positive men, a 763 bp region of gp120 (HXB2:6891–7654) was sequenced as follows. DNA was extracted from buffy-coats using a QIAamp DNA Mini Kit (Qiagen, Japan). The V3 region was PCR-amplified in accordance with a previously published primer and protocol [13]. The PCR products were further sequenced using a BigDye Terminator v3.1 Cycle Sequencing Kit (Applied Biosystems, USA) and an ABI Genetic Analyzer 3130 (Applied Biosystems, USA). The obtained sequence data were then analysed at the Infectious Diseases Surveillance Center of the National Institute of Infectious Diseases in Tokyo, Japan. Nucleotide sequences were aligned with 33 CRF01_AE sequences from Vietnam (GenBank accession numbers: FJ185228–FJ185260) analyzed in the previously cited report [2], 102 CRF01_AE sequences and 39 subtype reference sequences retrieved from the Los Alamos HIV Sequence Database (http://www.hiv.lanl.gov/) by MUSCLE [14]. Phylogenetic trees were inferred using the maximum likelihood (ML) method with an interior branch test by MEGA 6 [15]. For the ML tree inference, the study applied a general time reversible model with Gamma distributed and invariant sites to a base substitution model, based on model selection under the hierarchical likelihood test in MEGA 6.

HIV-1 co-receptor tropism was further predicted using the Geno2Pheno[co-receptor] (G2P) (http://coreceptor.bioinf.mpi-inf.mpg.de) based on the V3 sequence of HIV-1 *env*. To classify the tropism, 5.75% false-positive rates (FPR) were used as cut-offs, referencing European guidelines for tropism testing. Viruses with FPR below the cut-off were classified as cysteine-X-cysteine-chemokine receptor 4 usage (CXCR4, X4) or dual/mixed (DM) and viruses with FPR above the cut-off were classified as cysteine-cysteine-chemokine receptor 5 (CCR5, R5) usage [16].

### Data management and statistical analysis

Differences in men's baseline sociodemographic characteristics, risk behaviours and clinical characteristics as regards their wives' HIV status were evaluated using Pearson’s chi-squared test for categorical variables and the Wilcoxon rank-sum test for continuous variables. Reported sexual behaviours of husbands were compared to corresponding accounts given by their wives using Spearman’s correlation coefficient. To examine factors relating to wives’ HIV-positive status, univariate and multivariable logistic regression analyses were performed. Factors with potential correlation in univariate analysis (p<0.10) and clinically vital factors were included in the multivariable logistic regression model. The data was managed by EpiData 3.1 (EpiData Association, Denmark) and analysed by Stata version 12.0 (Stata Corporation, College Station, USA).

### Ethics

Standard HIV education and counselling was provided to all study participants. The study was approved by the BMH Ethical Committee and the Ethical Committee of the Institute of Tropical Medicine, Nagasaki University. Among eligible participants, only those who provided informed written consent were enrolled.

## Results

### Study participants

Of the 380 married men registered at the HIV clinic, 33 had died by the time of study enrolment, 45 had been referred to other hospitals, and 14 had gotten divorced. The remaining 288 men were invited to participate in the study. After providing informed consent and undergoing screening based on enrolment criteria, 163 men were enrolled in the study.

### Characteristics of HIV-1 positive married men

The study identified 101 men with HIV-negative wives (62%). Characteristics of HIV-positive married men are summarized in Table 1 and laid out according to HIV status of wives. Age at initial diagnosis did not significantly differ between men with HIV-positive wives and men with HIV-negative wives. Most couples from both groups had a substantial duration of marriage prior to HIV diagnosis, with a median duration of seven years. The duration of marriage prior to HIV diagnosis was longer among men with HIV-negative wives (p = 0.050). A total of 76% of men reported high-risk heterosexual behaviour such as sexual contact with FSWs as a possible transmission route. Half of men reported risky heterosexual contact as the only logical route, while one quarter reported both risky heterosexual contact and an IDU history. Another quarter of men reported IDU history as the only logical route. None of the men reported MSM as a logical route of transmission.

**Table 1:** Characteristics of HIV-positive married men.

			All		Men with HIV-positive wives		Men with HIV-negative wives		p-value
			N = 163		N = 62		N = 101		
Age at HIV diagnosis (year)			34	[30–39]	33.5	[29–36]	34	[31–39]	0.075
Marital duration before HIV diagnosis (years)			7.1	[3.7–13.1]	5.8	[2.1–10.4]	7.7	[4.5–13.7]	0.050
Possible risk of HIV transmission	Heterosexual contact		80	(49.1)	38	(61.3)	42	(41.6)	
	IDU		38	(23.3)	8	(12.9)	30	(29.7)	
	Both		39	(23.9)	15	(24.2)	24	(23.8)	
	No answer		6	(3.7)	1	(1.6)	5	(5.0)	0.031
Frequency of sex (times per month)	Before HIV diagnosis		4	[4–8]	4	[4–8]	4.5	[4–8]	0.645
	After HIV diagnosis		2	[1–4]	3	[1–4]	2	[1–4]	0.684
Frequency of unprotected sex (times per month)	Before HIV diagnosis		4	[3–8]	4	[3–8]	4	[3–8]	0.320
	After HIV diagnosis		0	[0–0]	0	[0–1]	0	[0–0]	< 0.001
History of genital ulcer	Men		28	(17.2)	14	(22.6)	14	(14.9)	0.152
	Their wives		48	(29.5)	27	(43.6)	21	(20.8)	<0.001
Clinical status at baseline	CD4+ T cell count (/μl)		62	[29–155]	90.5	[28–239]	55	[30–138]	0.240
	Viral load (log_10_ cp/ml)		5.18	[4.73–5.52]	5.00	[4.83–5.38]	5.23	[4.70–5.55]	0.481
	Clinical symptoms	Asymptomatic	25	(15.3)	17	(27.4)	8	(7.9)	
		Symptomatic	138	(84.7)	45	(72.6)	93	(92.1)	0.001
	HCV Ab		92	(57.5)	29	(47.5)	63	(63.6)	0.045
	HBsAg		31	(19.1)	16	(25.8)	15	(15.0)	0.089
ART status at enrolment	ART naïve		6	(3.7)	4	(6.5)	2	(2.0)	0.149
	Duration of ART (years)		0.9	[0–2.3]	0.9	[0–2.3]	0.8	[0–2.1]	0.990

Values are given as actual count (%) for categorical variables or median [inter-quartile] for continuous variables.

Intriguingly, an IDU history was significantly more common among men with HIV-negative wives than among men with HIV-positive wives (Table 1). Specifically, 70.1% of men with an IDU history had HIV-negative wives versus 52.5% of men without an IDU history (p = 0.042). Among men with an IDU history, behaviours such as sharing equipment, age at drug use initiation, duration of drug use, and current drug use practices did not appear to correlate with wives’ HIV status. Other factors such as men’s education and employment history also did not significantly correlate with wives’ HIV status (data not shown).

Unprotected sexual intercourse before HIV diagnosis was equally frequent among men with HIV positive wives and men with HIV negative wives. Almost 80% of men reported no condom use at all during sexual intercourse with their wives prior to HIV diagnosis. After HIV status was diagnosed and disclosed to wives, the proportion of men reporting condom use increased dramatically to 94.5%, though as can be expected the proportion was higher among couples learning of HIV-negative wives than among couples learning of HIV positive wives through screening (98.9% versus 87.7%, p<0.001). Crucially, there was no reported difference in the median [inter-quartile (IQR)] of the frequency of unprotected intercourse per month between men with an IDU history and men without, both before and after HIV diagnosis (4 [3–8] versus 4 [3–8], p = 0.537 and 0 [0–0] versus 0 [0–0], p = 0.559) as well as for the frequency of protected intercourse (4 [4–8] versus 4 [3–8], p = 0.664 and 3 [1–4] versus 2 [1–4], p = 0.297). Likewise, men with AIDS at the screening and men with asymptomatic HIV infection reported a similar frequency of unprotected intercourse per month before the diagnosis (4 [3–8] and 4 [4–6], p = 0.953). The group reporting the most frequent instance of unprotected intercourse before diagnosis was men with AIDS at screening and with HIV positive wives, 8 [3–10], although the difference was not significant (p = 0.953). Intercourse frequency reported by men in both groups was in line with corresponding accounts given by their wives. Reported condom use was also in line. For all couples, the Spearman correlation coefficient was 0.789 (p<0.001) and 0.875 (p<0.001) for frequency of reported unprotected sexual intercourse per month before and after HIV disclosure. The study also analysed sexual behaviour between couples with and without a desire to conceive. Men with the desire to conceive reported more frequent unprotected sexual intercourse than men without such a desire (6 [4–8] versus 4 [2–6], p = 0.003). Men with HIV-negative wives reported less desire to conceive than did men with HIV-positive wives (38.4% versus 56.5%; p = 0.025) and also had more existing children (p = 0.04).

A history of genital ulcers was more common among men with HIV-positive wives than among men with HIV-negative wives, though the difference was not significant. HCV infection is strongly associated with having an IDU history; HCV prevalence among IDU men was 93.4%, compared with 25% among non-IDU men (p<0.001). When HCV prevalence is used as a biomarker for the possible risk of parenteral transmission [17]; wives’ HIV status set against HCV prevalence among husbands and wives’ HIV status set against IDU history among husbands yielded similar proportions. Notably, the prevalence of HBsAg tended to be higher among men with HIV-positive wives than men without. HIV viral load data before ART initiation was available for 82 men (50.3%) and no difference was noted between men with HIV-negative wives and men with HIV-positive wives. Half the men in the study had progressed to AIDS at the first visit. Men with HIV-negative wives were significantly more likely to be symptomatic (p = 0.003). Lastly, there was no noteworthy difference in ART coverage and duration at time of enrolment between men in both groups (data not shown).

### Characteristics of wives

Of the wives of the 163 men enrolled in the study, 136 wives (58 HIV positive and 78 HIV negative wives) completed the questionnaire, while the remaining two HIV positive wives and 25 HIV negative wives did not. The majority of HIV positive wives (77.0%) were diagnosed within a month of their husbands’ diagnosis (median duration from husband to wife diagnosis of 4 days [IQR 0–29]). Median age at study enrolment was 31.3 years [IQR 28.3–36.8] with no significant difference in age between HIV-positive and HIV-negative wives (p = 0.18). Median age at HIV diagnosis was 28.4 years [IQR 25.6–36.2]. No HIV-negative wives reported an IDU or FSW history. Additionally, no HIV positive wives reported an IDU history, though one HIV-positive wife did report an FSW history. Twice as many HIV positive wives as HIV negative wives experienced genital ulcers, though this was not the case for men, where there was no significant difference between the two groups. Among HIV positive wives, there were three HCV Ab positive wives, three HBsAg positive wives, and one wife positive for HCV Ab and HBsAg. Most HIV positive wives had visited the hospital in asymptomatic stage (39, 69.6%). Median CD4+ T cell count was 307 cells/μl [IQR 212–419].

### Factors associated with having HIV-positive wives

Logistic regression analyses were performed to examine factors associated with men having HIV-positive wives (Table 2). The unadjusted analysis revealed the frequency of unprotected intercourse before HIV diagnosis of men did not significantly associate with having HIV-positive wives. As mentioned, men with an IDU history and men in the advanced stages of HIV infection were less likely to have HIV-positive wives. By contraries, a history of genital ulcers in wives positively associated with men having HIV-positive wives. No association was found with ART duration and CD4+ T cell count.

**Table 2:** Risk factor analysis for men having HIV-positive wives.

		Crude OR (95%CI, *P*-value)	Adjusted OR (95%CI, *P*-value)
Age at HIV diagnosis (every 10 years)		0.76 (0.50–1.15, 0.190)	0.64 (0.36–1.11, 0.110)
Duration of ART before enrolment (year)		1.09 (0.69–1.71, 0.726)	1.44 (0.82–2.52, 0.200)
Duration from marriage to HIV diagnosis (year)		0.97 (0.92–1.01, 0.103)	
Monthly frequency of unprotected sex before HIV diagnosis of men		1.05 (0.97–1.15, 0.231)	1.11 (0.99–1.24, 0.083)
History of genital ulcers	Husband	1.82 (0.80–4.12, 0.155)	
	Wife	2.14 (1.05–4.38, 0.037)	2.17 (0.91–5.22, 0.082)
Risks of HIV transmission	Heterosexual contact	1.0	1.0
	IDU	0.29 (0.12–0.72, 0.007)	0.31 (0.10–0.95, 0.041)
	Both	0.69 (0.32–1.50, 0.353)	0.69 (0.25–1.85, 0.454)
CD4+ T cell count at baseline (every 100/μl)		1.14 (0.92–1.42, 0.224)	
Clinical symptoms at baseline	Asymptomatic	1.0	1.0
	Symptomatic	0.23 (0.09–0.57, 0.001)	0.20 (0.06–0.75, 0.016)
Positive HBsAg		1.97 (0.89–4.35, 0.092)	3.12 (1.08–9.00, 0.035)
Positive HCV Ab		0.52 (0.27–0.99, 0.047)	

In the multiple analysis, the model was adjusted for age at HIV diagnosis, duration of ART before study enrolment, couple’s frequency of unprotected intercourse before the HIV diagnosis of the man, history of genital ulcers in wives, risk of HIV transmission, existence of clinical symptoms, and serostatus of HBsAg. HIV viral load was not added to the model due to the limited nature of available data. Negative associations with IDU history (adjusted OR 0.31, p = 0.041) and advanced stage of infection (adjusted OR 0.20, p = 0.035) as regards having HIV-positive wives remained significant, even after adjustments. The frequency of unprotected intercourse before the diagnosis of men remained insignificant and a history of genital ulcers in wives turned out to be insignificant in the multiple analysis. Interestingly, the association between the HBsAg positivity and having HIV-positive wives became significant after adjusting for the multiple model. Due to the strong link between HCV Ab and IDU history, the model includes only IDU history. However it is worth mentioning similar results were obtained when using HCV Ab as an indicator for IDU history; each can often be considered a proxy for the other. When the analysis was conducted with clinical symptoms categorized into the three groups (asymptomatic, symptomatic non-AIDS, AIDS), the model displayed similar results (data not shown).

### Sequence analysis

Of the 163 buffy-coat samples, the V3 sequence from 80 samples was successfully amplified and identified as CRF01_AE (accession number: KP401976–HP401985, KP401987–KP402056). Further phylogenetic analysis (S1 Fig) revealed an evolutionary relationship between our samples and an epidemic history proposed in the previous report [2]. Sixty-nine sequences were clustered with the cluster 2+3 in the previous report [2], which contained strains derived from IDUs in Vietnam. Six sequences formed a sub-cluster, which corresponded to the northern Vietnam/Guangxi IDU cluster (cluster 3). The remaining 11 sequences were paraphyly connected to the IDU cluster and were evolutionary related to heterosexual individuals in southern Vietnam and Thailand.

Notably, 34 out of 69 samples (49.3%) in the Vietnam IDU cluster were taken from IDU men, and 48 out of 69 were taken from men with HIV-negative wives, with considerable overlap of 25 samples. The remaining 11 samples contained seven from IDU men and six from men with HIV-negative wives. Co-receptor tropism was predicted in the 80 sequenced viruses: 9 could not be classified, 44 (55.0%) were deemed CXCR4 with the 5.75% cut-off by G2P. Of the 71 men with determined HIV tropism, 46 had HIV negative wives and 32 out of 46 (69.6%) had CXCR4-tropic strain. Additionally, 12 (48.0%) out of 25 men with HIV positive wives had CXCR4-tropic strain.

### Follow up of HIV serodiscordant couples

Of the 101 serodiscordant couples enrolled in 2012, the team followed up 61 couples in 2014. In total, 126.5 person-years of observation were carried out (median observation period 24.1 months, range 13.1–34.6 months). All the men had been on ART. Follow-up HIV screening found no wives had seroconverted. The median frequency of sexual intercourse was 2 times per month during the observation year; 10 couples (16.4%) reported inconsistent condom use and 2 couples (3.3%) reported no condom use. Frequency of intercourse after HIV diagnosis as reported in the follow-up survey did not differ from the frequency reported in the initial survey, also post diagnosis (2 versus 2 times per month, p = 0.868). There was an increase in the percentage of men admitting not always using a condom during intercourse, from 5.6% to 16.4%, though in context the difference was not statistically significant (p = 0.089).

## Discussion

### High serodiscordance in northern Vietnam

This is the first study to investigate HIV-affected married couples in Vietnam with injecting drug use as a significant transmission route among men. To our surprise, HIV serodiscordance was as high as 62%, and particularly high among couples where the man’s viral sequence belonged to the Vietnam IDU cluster (69.0%). This is despite the fact most couples had had a long duration of marriage with a considerable amount of unprotected sexual contact. This level of serodiscordance was notably higher than in previously published reports, including 24–31% from hospital studies in Thailand (where CRF01_AE is epidemic) and 42% and 55% from cross-sectional hospital studies in South India and Brazil, respectively [18–20].

As we analysed risk factors for HIV transmission among married couples, we found couples where the man had an IDU history still displayed a significantly high level of serodiscordance. To our knowledge, this association has not been previously demonstrated. It appears independent from sexual behaviour and other risk factors, such as genital ulcers and the use of ART. Although men with HIV positive wives had engaged in more unprotected sex after the HIV diagnosis of men than men with HIV negative wives, most wives were deemed to have seroconverted before the HIV diagnosed of men, and not during the duration between diagnosis of men and that of their wives. In most cases, the HIV diagnosis of wives was made immediately after the diagnosis of their husbands, and men had developed a more advanced clinical stage at the HIV diagnosis. Therefore, sexual behaviour after the HIV diagnosis of men was not deemed a risk factor in our setting. The above finding may indicate injecting drug use as a predominant mode of blood-borne transmission may be linked to the high serodiscordance among Vietnamese couples. Furthermore, the phylogenetic analysis demonstrated the dominance of IDU-associated HIV strains among the study population, regardless of whether drug use was linked to transmission. This may explain why the proportion of HIV-positive wives was smaller even among men with only heterosexual transmission risk than among married men in northern Thailand, also with heterosexual contact as the main transmission risk [19]. The dominance of HIV serodiscordance or lower seroconversion rate among stable heterosexual couples in which the man was infected by parenteral transmission has been observed in other reports. For instance, the three primary types of HIV epidemic in China have been blood-donor centred in Henan, IDU centred in Yunnan, and sexual-transmission centred in other areas; of these the Henan and Yunnan epidemics have a lower seroconversion rate among HIV serodiscordant couples [21], though it should be noted the study did not systematically analyse HIV risk factors as well as transmission efficacy. A multi-centre cohort study of HIV-positive men with haemophilia in the United States reported only 13% seroconcordance [22]. Lower seroconcordance (higher discordance) among heterosexual couples in which the male partner had an IDU history was also reported in Thailand, though the study attributed this difference to the effect of subtype differences; IDU men were infected with subtype B whereas non-IDU men were infected with subtype CRF01_AE [18]. Taken with the results of our study, these findings suggest prior mode of transmission may contribute to subsequent transmission efficacy. Therefore we hypothesize that HIV strains derived from the IDU population in northern Vietnam transmit less efficiently through sexual contact than do other strains. On the other hand, an IDU-associated virus already transmitted through heterosexual contact may regain some of this transmission efficacy. Though it is difficult to speculate on mechanisms that might bring about this potential difference, some unique characteristics of HIV strains in Vietnam, such as the dominance of CXCR4 tropism observed in our study or a previous report [23], may have contributed to the apparent lower sexual transmission efficacy among stable couples in northern Vietnam. However, only half of the HIV *env* sequences could be successfully evaluated, and sequence data of the V3 loop alone cannot determine co-receptor tropism of CRF01_AE stains [24]. Therefore, there was insufficient data to discuss the relationship between viral factors and the transmission efficacy. In addition, there are several limitations in our study. First, behavioural data relies on self-reporting, making it difficult to avoid recall bias and social desirability bias. In this case, however, we believe our behavioural data is meaningful, as there was strong concordance between the desire to conceive and the frequency of sexual intercourse, as well as between answers obtained from men and the corresponding answers obtained from their wives. The second is the limited number of available viral load data due to infrastructural limitations. The third is the lack of virological confirmation regarding intra-couple transmission. The fourth is the risk factor analysis was done in the form of a cross-sectional design, meaning we could not discuss causality and were forced to exclude cases in which the man had died before the enrolment period. Taken together, unmeasured confounding factors might be underlying the results; the further investigation is required to discuss the possible risk factors among this population.

There are other potential influences that should be taken into consideration. One concerns host factors; CCR5 mutation, high secretion of beta chemokines, or killer immunoglobulin-like receptor (KIR)/HLA combination are well known to influence HIV transmission [25]. Although CCR5 delta 32 mutation is rare among Asian populations, two possible protective CCR5 mutations were reported in highly exposed persistent seronegative (HEPS) Vietnamese IDUs [26]. High Natural Killer (NK) cell activity [27], enhanced CD4+ T cell response to beta chemokines, and inhibition of post-entry viral replication [28] were also observed in these groups. Some wives in the study may also harbour these protective genetic factors, and this should be considered when trying to explain the relative lack of HIV transmission. However, as the study population was drawn from a single ethnic group with relatively uniform genetic distribution, host factors such as these would be unlikely to account for the full lack of HIV transmission efficacy. Another influence concerns risk behaviours of wives. Wives reported few risk behaviours save for those associated with having HIV positive husbands. Although a small number of wives tested positive for hepatitis B and/or hepatitis C co-infection implying the presence of past risk behaviours, the overall impact would likely not be considerable. The result of the survey of wives is also consistent with a published review of the HIV epidemic among Vietnamese women [9]. Therefore among our study population, the behaviour of wives would be highly unlikely to account for any meaningful difference.

### Advanced HIV infection at the time of diagnosis

Among the study population, HIV diagnosis in the advanced stage was negatively associated with HIV transmission to wives. This finding is compatible with that of a previously published hospital-based study in Thailand [19], though it does contradict the widely accepted understanding that viral load plays a key role in HIV transmission as outlined in several large population based studies [29,30]. We believe this discrepancy can be attributed in part to the hospital-based setting. Our study and the study in Thailand were both conducted at referral hospitals where most patients were symptomatic with a low CD4+ T cell count and with HIV-negative spouses that had been exposed to the virus for a substantial amount of time. It is also plausible that men at an advanced stage of infection had less sexual intercourse with their wives at this time because they were feeling unwell, and this could explain the negative association with HIV transmission. However, there was no difference in sexual behaviors according to clinical status of men at the baseline.

### Co-infection with HBV

Interestingly, there was a significant positive association between HBV co-infection among index men and HIV transmission to wives. Although there have been reports showing high mortality among HIV-positive patients with HBV co-infection in Thailand [31] and the United States [32], to our knowledge HBV co-infection has not been found to enhance the risk of HIV sexual transmission. According to a previously published in vitro study, HBV-X protein super-induces on-going HIV replication and HIV-1 long-terminal repeat transcription [33]. Although no studies known to us have shown HBV co-infection to increase the HIV viral load, HIV replication in the genital area may have been further activated in HBV-co-infected men, and this in turn may have facilitated transmission.

### Lack of seroconversion in the follow-up study

We found no seroconversion after the median 24.1 months of follow-up, despite some degree of unprotected intercourse and a downward tendency in condom use. This was in contrast to the results of studies conducted in the pre-ART era, which showed a substantial seroconversion rate (3.0–12.0/100 person-years) [19,34–37]. This difference is likely explained by high ART coverage reducing the transmission risk from index men [30], as well as better condom use. Although substance abuse is considered related to poor treatment adherence [38], our results imply proper education and treatment can lead to successful prevention of sexual transmission in long-term relationships where substance abuse is an issue. Diagnosing HIV in the early stages and providing counselling and treatment is now the cornerstone of the HIV prevention effort. Our results endorse the importance of screening both partners making up HIV affected couples and actively introducing early ART interventions.

## Conclusions

This is the first study comprehensively investigating HIV-affected married couples in Vietnam. The proportion of HIV serodiscordance was high, particularly among couples where the HIV-infected man had an IDU history, and in both cases despite substantial sexual risk behaviours before the diagnosis. A relatively high number of at-risk wives therefore remain HIV-negative and can still be protected if couples are made aware of this serodiscordance through screening. As our study is likely indicative of the wider population, such screening could lead to the prevention of eventual transmission to wives. Longitudinal follow-up demonstrated no seroconversion among HIV-serodiscordant couples where HIV-positive men had received ART. Taken as a whole, our study re-emphasizes the importance of active HIV screening and early ART provision to couples.

## Supporting Information

S1 Fig Evolutionary relationships of gp120 sequences from 80 men. The phylogeny was inferred using the Maximum Likelihood method and the tree with the highest log likelihood (-28676.0757) is shown. Sequences belonging to other subtype than CRF01_AE were omitted after the inference and represented a branch connected to the tree-root. Bootstrap probability with >80% was indicated for each cluster. Red open square, red open circle, and red circle indicated virus samples from heterosexual men, IDU men, and men with both risk in our subjects, respectively. Black square, black open circle, and black circle showed reference sequences in the previous report [2] belonging to cluster 1 (southern Vietnam heterosexual individuals), cluster 2 (southern Vietnam IDU individuals), and cluster 3 (northern Vietnam or Guangxi IDU individuals), respectively. Dotted line highlights CRF01_AE references retrieved from HIV sequence database. Grey and brown hatching showed the sample clusters corresponding to cluster 2 and 3, respectively.
(PDF)
