# Supplementary material for: High Proportion of HIV Serodiscordance among HIV-Affected Married Couples in Northern Vietnam
Source: PLoS One. 2015 Apr 21;10(4):e0125299. doi: 10.1371/journal.pone.0125299 (PMC4405585; doi:10.1371/journal.pone.0125299)

**S1 Figure. Evolutionary relationships of gp120 sequences from 80 men.**

The phylogeny was inferred using the Maximum Likelihood method and the tree with the highest log likelihood (-28676.0757) is shown. Sequences belonging to other subtype than CRF01_AE were omitted after the inference and represented a branch connected to the tree-root. Bootstrap probability with >80% was indicated for each cluster. Red open square, red open circle, and red circle indicated virus samples from heterosexual men, IDU men, and men with both risk in our subjects, respectively. Black square, black open circle, and black circle showed reference sequences in the previous report [2] belonging to cluster 1 (southern Vietnam heterosexual individuals), cluster 2 (southern Vietnam IDU individuals), and cluster 3 (northern Vietnam or Guangxi IDU individuals), respectively. Dotted line highlights CRF01_AE references retrieved from HIV sequence database. Grey and brown hatching showed the sample clusters corresponding to cluster 2 and 3, respectively.

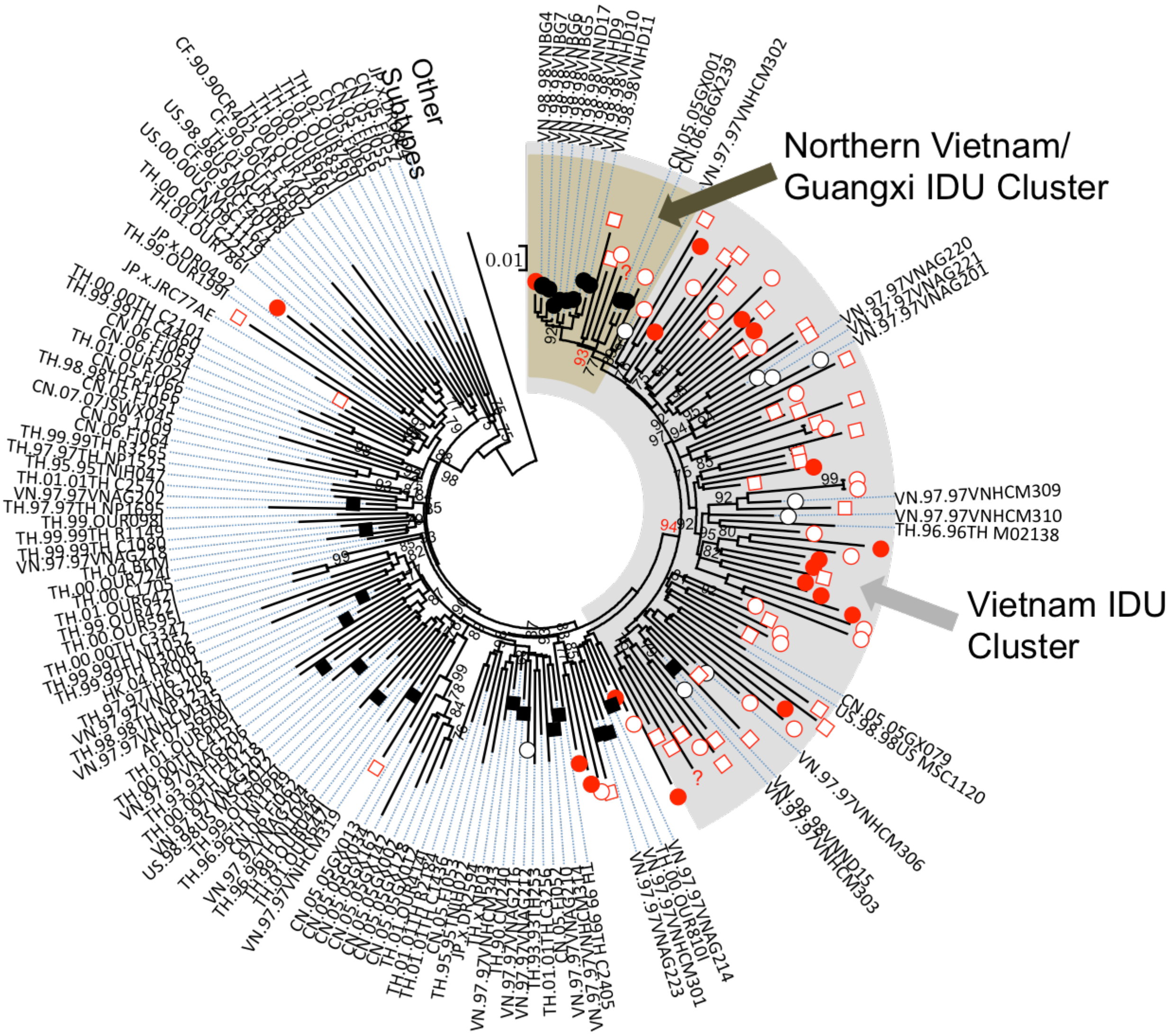

Supplement: S1 Fig — The phylogeny was inferred using the Maximum Likelihood method and the tree with the highest log likelihood (-28676.0757) is shown. Sequences belonging to other subtype than CRF01_AE were omitted after the inference and represented a branch connected to the tree-root. Bootstrap probability with >80% was indicated for each cluster. Red open square, red open circle, and red circle indicated virus samples from heterosexual men, IDU men, and men with both risk in our subjects, respectively. Black square, black open circle, and black circle showed reference sequences in the previous report [2] belonging to cluster 1 (southern Vietnam heterosexual individuals), cluster 2 (southern Vietnam IDU individuals), and cluster 3 (northern Vietnam or Guangxi IDU individuals), respectively. Dotted line highlights CRF01_AE references retrieved from HIV sequence database. Grey and brown hatching showed the sample clusters corresponding to cluster 2 and 3, respectively. (PDF) [file pone.0125299.s001.pdf]
